# Seawater drowning-associated pneumonia: a 10-year descriptive cohort in intensive care unit

**DOI:** 10.1186/s13613-017-0267-4

**Published:** 2017-04-26

**Authors:** Alexandre Robert, Pierre-Éric Danin, Hervé Quintard, Nicolas Degand, Nihal Martis, Denis Doyen, Céline Pulcini, Raymond Ruimy, Carole Ichai, Gilles Bernardin, Jean Dellamonica

**Affiliations:** 1 Service de Réanimation Médicale, Hôpital l’Archet 1, CHU de Nice, 151 Route Saint Antoine de Ginestière, CS 23079, 06202 Nice Cedex 3, France; 2 Service de réanimation polyvalente, Hôpital l’Archet 2, CHU de Nice, 151 Route Saint Antoine de Ginestière, CS 23079, 06202 Nice Cedex 3, France; 3 Unité INSERM 1065 Team 8, Laboratoire C3M, Hôpital l’Archet 2, Université Côte d’Azur, 151 Route Saint Antoine de Ginestière, CS 23079, 06202 Nice Cedex 3, France; 4 Service de réanimation polyvalente, Hôpital Pasteur 2, CHU de Nice, 30 Voie Romaine, CS 51069, 06001 Nice Cedex 1, France; 5 Institut de Pharmacologie Moléculaire et Cellulaire, Centre National de la Recherche Scientifique (CNRS), Université Côte d’Azur, 660 Route des Lucioles, 06560 Valbonne, France; 6 Service de Microbiologie, Hôpital l’Archet 2, CHU de Nice, 151 Route Saint Antoine de Ginestière, CS 23079, 06202 Nice Cedex 3, France; 7 Service de Médecine Interne, Hôpital l’Archet 1, CHU de Nice, 151 Route Saint Antoine de Ginestière, CS 23079, 06202 Nice Cedex 3, France; 8 CHRU de Nancy, Service de Maladies Infectieuses et Tropicales and Université de Lorraine, EA 4360 APEMAC, Nancy, France; 9 Unité INSERM 1065 Team 6, Laboratoire C3M, Hôpital l’Archet 2, Université Côte d’Azur, 151 Route Saint Antoine de Ginestière, CS 23079, 06202 Nice Cedex 3, France; 10 IRCAN, Faculté de Médecine, Université Côte d’Azur, 06000 Nice, France; 11 Unité INSERM 1065 Team 3, Laboratoire C3M, Hôpital l’Archet 2, Université Côte d’Azur, 151 Route Saint Antoine de Ginestière, CS 23079, 06202 Nice Cedex 3, France

**Keywords:** Drowning, Seawater, Pneumonia, Antibiotic, Resistance

## Abstract

**Background:**

Pneumonia is one of the major complications of drowning, but the optimal empirical antibiotic treatment is not clearly defined. Multidrug-resistant (MDR) bacteria and fungi have been identified in a recent series of freshwater drowning-associated pneumonia. However, microbial data in seawater drowning are scarce. The objective of the study is to describe the microorganisms isolated in early respiratory specimens obtained from seawater drowning-associated pneumonia and to provide their antibiotic susceptibility pattern.

**Methods:**

All patients admitted for seawater drowning between 2003 and 2013 to two intensive care units, from the region in France with the highest drowning rate, were retrospectively included. Demographics, antimicrobial therapy and microbiological data from respiratory samples collected within the first 48 h after admittance were analyzed.

**Results:**

Seventy-four drowned patients were included, of which 36 (49%) were diagnosed by the clinician as having early pneumonia. Concerning the overall population, the median simplified acute physiology score (version 2) was 45 (30–65), and the mortality was 26%. Twenty-four respiratory samples from different patients were obtained within the first 48 h. Sixteen were positive. The main microorganisms found were *Enterobacteriaceae* (*Enterobacter* spp., *Klebsiella* spp. and *Escherichia coli*) and Gram-positive aerobic cocci (*Streptococcus pneumonia* and *Staphylococcus aureus)* with a low rate of antimicrobial resistance. No MDR bacteria or fungi were identified. However, among the positive respiratory samples collected, 5/16 (31%) grew bacteria with natural resistance to amoxicillin–clavulanate, the first-line antibiotic commonly used in our cohort. Resistance was only found among Gram-negative bacteria and from respiratory samples of patients with a higher drowning grade at admission (*p* = 0.01).

**Conclusions:**

This 10-year descriptive study, the largest cohort to date, provides early respiratory samples from seawater drowning patients. The microorganisms retrieved were either mostly part of the human oro-pharyngeal flora or *Enterobacteriaceae* and displayed low rates of antimicrobial resistance. Respiratory samples should nonetheless be collected at admittance to the ICU to avoid inappropriate treatment. Empiric use of cephalosporin could be restricted to severe patients or if Gram-negative bacilli are found after direct examination.

## Background

Despite recent progress in the management of respiratory complications due to drowning [1, 2], criteria to initiate antibiotic or to collect respiratory samples are lacking [2–4]. In addition, available data on the microorganisms involved in drowning-associated pneumonia are too scarce to recommend a straightforward antibiotic strategy [3]. Two recent series on freshwater drowning in urban areas, from Paris and Amsterdam region with, respectively, 37 and 49 patients [5, 6], described a significant proportion of MDR bacteria or presence of fungi in early respiratory samples suggesting the use of broad-spectrum antibiotics or even empirical antifungal therapy. Water composition (i.e., freshwater or seawater) and locality may influence such susceptibility patterns [2]. However, to the best of our knowledge, microbial data in seawater drowning-associated pneumonia are very scarce and generally gathered from cases reports [7–10] or reviews of cases from warm seas [3] but none from temperate Mediterranean zones.

The aim of this study is to describe the bacterial species and their antibiotic susceptibility patterns recovered from early respiratory samples of drowning-associated pneumonia from seawater drowning patients in the Mediterranean Sea.

## Methods

### Study population and demographics

This retrospective observational bi-center study traced and identified through ICD coding of all adult patients admitted for seawater drowning between 2003 and 2013 in two intensive care units in the city of Nice (Mediterranean Sea) which lies in the region with the highest drowning rate in France [11]. Patients admitted for swimming pool or freshwater drowning were excluded. There were no specific guidelines for the management of drowning pneumonia in either center.

Drowning was defined as respiratory impairment following immersion in water, whatever the survival [12]. The drowning grade was determined according to the recent classification described by Szpilman et al. [2, 13] (Table 1). Collected data were: age, sex, presence of initial cardiac arrest, drowning grade [2], respiratory samples collected within 48 h, microorganisms and antibiotic susceptibility patterns, diagnosis of pneumonia or ARDS, causes of drowning, season and place of drowning, duration of mechanical ventilation, length of stay and mortality in the ICU, therapeutic administration and the SAPS2 score [14].

**Table 1:** Drowning grade derived from Szpilman et al. [2, 13]

Grade	Initial clinical description
1	Answer to verbal or tactile stimuli with normal pulmonary auscultation but cough
2	Answer to verbal or tactile stimuli with rales in some pulmonary fields
3	Answer to verbal or tactile stimuli with rales in all pulmonary fields without hypotension or shock
4	Answer to verbal or tactile stimuli with rales in all pulmonary fields and hypotension or shock
5	No answer to verbal or tactile stimuli with presence of pulse
6	No answer to verbal or tactile stimuli without presence of pulse and less than one hour of Immersion and no obvious physical evidence of death

Because of the observational nature of the study, patient’s consent was waived. Follow-up for the study was performed until ICU discharge. According to the Commission Nationale de l’Informatique et des libertés, there were no needs of specifics authorization because of the anonymous character of the data collected.

### Bacteriology and microbiological data

Respiratory samples collected within the first 48 h of admission were analyzed. Cultures were incubated in aerobic (with or without 5% CO_2_) and in anaerobic conditions during 48 h at 35 °C on both selective and non-selective media. Colonies of interest were identified, quantified and tested for antibiotic susceptibility. The 48-h limit was retained so as to exclude ICU-acquired infection (i.e., ventilator-associated pneumonia) [15].

In accordance with current guidelines, the quantitative bacterial thresholds used to define bacterial pneumonia were 10^3^ colony-forming units per milliliter (cfu/mL) for distal protected samples and 10^5^ cfu/mL for tracheal aspirates [16]. When tracheal aspirates were performed, only high-quality specimens (i.e., with more than 25 neutrophils and less than 25 epithelial cells per microscopic field) graded groups 4 or 5 according to the Murray–Bartlett–Washington definition were taken into consideration [17].

### Diagnosis of pneumonia

Diagnosis of pneumonia was based on miscellaneous criteria, including fever defined by body temperature of more than 38.5 °C, presence of purulent endotracheal secretions, leukocyte blood count greater than 10,000/mm^3^ or less than 4000/mm^3^, and radiological features of pneumonia according to the perception of the clinician [18–20]. Bacteriological criteria could not always be used because respiratory samples were not systematically collected. Diagnosis of ARDS was made according to the 2012 Berlin definition [22] and based on the American and European Consensus Conference on ARDS prior to 2012 [23].

### Statistical analysis

Statistical analyses were performed using the Statistical Package for Social Science software, version 19 (SPSS, Chicago, Illinois, USA). Continuous variables are presented as median and 25th–75th percentile. Categorical variables are presented as either rates or percentages. The Fisher’s exact test was used for comparison of categorical variables. U Mann and Whitney tests were used for comparison of continuous variables. Differences were considered statistically significant when the *p* value was <0.05.

## Results

### Demographics

From January 2003 to April 2013, 74 patients were admitted for seawater drowning to the two ICU. None of them was excluded.

The male-to-female sex ratio was 1.85, and the median age was 68 (51–77) years. Thirty patients (41%) presented out-of-hospital cardiac arrest. Nineteen patients died during their ICU stay, defining a 26% ICU mortality rate. The median SAPS2 score was 45 (30–65). The main objectified causes for drowning were medical with 22/74 (30%) cases of divers medicals pathologies (such as seizures or cardiovascular dysfunction). Other causes were 16 (22%) sports-related injuries or unspecified trauma (including those occurring under the influence of alcohol), 7 (9%) physical exhaustion and 6 (8%) suicides. Unknown causes accounted for 23 (31%) cases in which resuscitation was carried out without prior knowledge of the time of drowning. Patient characteristics are summarized in Table 2.

**Table 2:** Demographics and clinical characteristics of the patients

Characteristic	*N* = 74
At admission	
Age, years	68 (51–77)
Male sex—no. (%)	48 (65)
Initial cardiac arrest—no. (%)	30 (41)
Drowning grade	5 (3–6)
In the ICU	
Early pneumonia—no. (%)	36 (49)
Patients receiving antibiotics during the first 48 h—no (%)	44 (59)
Respiratory samples collected within the first 48 h—no. (%)	24 (32)
Positive respiratory samples collected within the first 48 h—no. (%)	16 (22)
Pneumonia treated empirically with AMC—no. (% of 36)	34 (94)
Appropriate empirical treatment within the first 48 h—no. (% of 16)	8 (50)
ARDS—no. (%)	25 (34)
SAPS2 score	45 (30–65)
Invasive mechanically ventilated patients—no. (%)	51 (69)
Duration of mechanical ventilation, days	1 (0–4)
Length of stay, days	3 (2–7)
Mortality rate in the ICU—no. (%)	19 (26)

Continuous variables are expressed as medians with their interquartile range in brackets; categorical variables are presented within brackets as percentages

*ICU* intensive care unit, *ARDS* acute respiratory distress syndrome, *AMC* amoxicillin–clavulanate

All patients were found at sea in a similar coastal environment within 300 meters of the coast. In 61 (84%) cases, drowning occurred during the summer and spring.

### Pneumonia, respiratory samples and bacteriological data

Of the 74 patients, four died within the first 24 h of admission and before any bacteriological sample could be obtained. Thirty-six patients (49%) were diagnosed and curatively treated for pneumonia. Twenty-five (34%) had ARDS. Four patients (5%) received curative antibiotics (more than 24 h) without proven pneumonia diagnosis and without respiratory sample during the first 48 h. Four additional patients (5%) received antibiotics for less than 24 h after admission, until the medical team challenged the diagnosis of pneumonia. Finally, 44 patients (59%) received antibiotics; among them, 40 (54%) received antibiotics for more than 24 h during the first week of hospitalization.

Respiratory specimens were obtained from 24 (32%) patients within the first 48 h of admission to the ICU with 19 (79%) protected distal samples and 5 (21%) tracheal aspirates. Bacteria were isolated at a significant count from 16 respiratory samples (22% of all patients and 67% of the early respiratory samples), and 24 different bacterial species were identified. *Enterobacter* spp. were the most frequent microorganisms followed by *Streptococcus pneumonia* and *Staphylococcus aureus. Pseudomonas aeruginosa* was found in only one case. No fungi were isolated (Table 3).

**Table 3:** Identification of microorganisms isolated from respiratory samples

Microorganism	*N* = 24
Aerobic Gram-negative bacilli	14 (58%)
*Enterobacter cloacae*	3^a^
*Enterobacter aerogenes*	2^a^
*Escherichia coli*	2
*Citrobacter koserii*	2
*Klebsiella pneumoniae*	1
*Klebsiella oxytoca*	1
*Haemophilus influenzae*	2
*Pseudomonas aeruginosa*	1^a^
Gram-positive aerobic cocci	10 (42%)
*Streptococcus pneumoniae*	4
*Staphylococcus aureus*	3
Indefinite oral flora	3

*N* The total number of positive cultures of patients

^a^Resistance to amoxicillin-clavulanate

Antibiotic susceptibility testing showed chromosomal penicillinase in four cases (for two *Klebsiella* spp. and two *Citrobacter koserii*) with a preserved susceptibility to amoxicillin–clavulanate (AMC). An inducible chromosome-encoded AmpC cephalosporinase was present in six strains: five *Enterobacter* spp. and one *Pseudomonas aeruginosa*. *P. aeruginosa* isolate is also resistant to ticarcillin, ticarcillin–clavulanate and aztreonam due to overexpression of MexAB-OprM efflux pump (Table 4) but did not qualify as a multidrug-resistant (MDR) bacteria according to international criteria [21]. Five respiratory samples had at least one strain with bacterial resistance to AMC. Overall, resistance to AMC was observed in 5/16 (31%) of early respiratory samples and in 6/24 (25%) bacterial species (Table 3). All cases of AMC resistance were observed for the Gram-negative bacilli species with 6/14 (43%) resistant.

**Table 4:** Mechanisms of betalactamins resistance in strains

Resistance mechanisms	*N* = 13
Natural resistance	10 (77%)
Chromosomal penicillinase: *Klebsiella* spp./*Citrobacter koserii*	4
AmpC cephalosporinase: *Enterobacter* spp./*Pseudomonas aeruginosa*	6
Acquired resistance	3 (23%)
MexAB-OprM efflux pump: *Pseudomonas aeruginosa*	1
Inducible penicillinase: *Staphylococcus aureus*	2

*N* Total of identical resistance mechanism found in all of samples

### Characteristics and outcomes of patients according to the resistance of the microorganisms

Compared to patients with sensitive microorganisms, patients with naturally resistant microorganism to AMC, found during the first 48 h, had a higher rate of cardiac arrest (*p* = 0.026), higher drowning grade (*p* = 0.013), longer duration of initial resuscitation (*p* = 0.004), longer duration of mechanical ventilation (*p* = 0.025) and a higher mortality rate in ICU (*p* = 0.013). The SAPS2 score was not significantly different between the two groups nor the age, sex, initial Glasgow score, number of comorbidities (such as COPD, diabetes, coronary disease and chronic kidney disease), rate of invasive ventilation and length of stay (Table 5).

**Table 5:** Clinical characteristics and outcomes of patients with positives respiratory samples according to the sensitivity to amoxicillin-clavulanate of the microorganisms found

	AMC S *N* = 11	AMC R *N* = 5	*p* value
Characteristics at admission			
Age, years	53 (44–72)	68 (60–71)	0.571
Male sex—no. (%)	6 (55)	3 (60)	1
Initial cardiac arrest—no. (%)	3 (27)	5 (100)	0.026
Low flow, minutes	0 (0–1)	8 (5–10)	0.004
Drowning grade	5 (4–6)	6 (6–6)	0.013
Initial Glasgow score	4 (3–11)	3 (3–3)	0.051
SAPS2 score	45 (37–52)	65 (65–66)	0.069
Various comorbidities (COPD, diabetes, coronary disease, kidney malignancy)—no. (%)	3 (27)	2 (40)	1
Outcome			
Invasive mechanically ventilated patients—no. (%)	10 (91)	5 (100)	1
Duration of mechanical ventilation, days	2 (1–5)	14 (6–19)	0.025
Length of stay, days	4 (2–6)	7 (6–19)	0.3
Mortality rate in the ICU—no. (%)	1 (9)	4 (80)	0.013

Continuous variables are expressed as medians with their interquartile range in brackets; categorical variables are presented within brackets as percentages

*ICU* intensive care unit, *COPD* chronic obstructive pulmonary disease, *AMC* amoxicillin–clavulanate

### Type of antibiotic therapy and adaptation to the microbiological results

AMC was the most common empiric antimicrobial treatment within the first 48 h (in 34/36 cases, 94%). However, two patients received, on admission, piperacillin–tazobactam as empiric treatment that was downgraded to AMC after obtaining the results of antibiotic susceptibility testing. Thirty-three patients received AMC as definitive treatment, and another three received cephalosporin for infection due to *Enterobacter* spp. The median duration of the antibiotic treatment was 7 (5–7) days.

All patients with positive respiratory specimens were initially treated empirically, 8/16 (50%) had appropriate empirical treatment, 4/16 (25%) had their treatment modified after receiving the bacteriological results (adapted to culture and susceptibility testing), 3/16 (19%) did not benefit from adaptation of their treatment to the microbiological results without a clear explanation mentioned in the medical record, but survived in the ICU. One patient died in the ICU without adaptation of the antibiotic treatment because the decision to limit life-sustaining therapies was taken before obtaining the final bacteriological results.

## Discussion

This 10-year retrospective study is to date the largest cohort of early respiratory sample collection from seawater drowning patients. We found that wild-type *Enterobacter* spp. is the most represented bacterial species and is likely due to aspiration of contaminated water or endogen flora. Among the respiratory samples collected, 5/16 (31%) grew bacteria with natural resistance to amoxicillin–clavulanate, the first-line antibiotic commonly used in our cohort. Unlike freshwater drowning-associated pneumonia, no specific MDR bacteria nor fungi were found [5, 6].

Pneumonia appears to be a common complication in large series of drowning survivors admitted to ICU [4] and was diagnosed in nearly half of all patients in our series. Antimicrobial susceptibility patterns are currently debated as illustrated in recent series of freshwater drowning in urban areas. Low rates of antibiotic resistance were found in respiratory samples from our patients. Only one strain of *Pseudomonas aeruginosa* was found, and despite showing resistance to ticarcillin, it did not qualify as a MDR species [21].

However, resistance to AMC was seen in a third of cases mainly due to *Enterobacter* spp. This chromosomal resistance pattern could not be associated with a specific population in our study, but these findings should encourage clinicians to perform respiratory sample collection as early as possible to avoid inappropriate antibiotic therapy.

We cannot identify the pathophysiological or environmental processes that would explain the microbial findings. The bacterial species that were isolated are likely to come from the oro-pharyngeal and/or gastric flora and would suggest aspiration pneumonia as described in previous series [22]. Conversely, the large proportion of *Enterobacter* spp. that was identified in our series is not typical of aspiration pneumonia and suggests seawater inhalation.

Accidental bacterial flora constituted by fecal contamination has been frequently described in seawater [23], and this contamination could explain the bacterial findings. French local authorities are therefore frequently compelled to specifically quantify this flora as a marker of marine pollution, but their analysis mainly focuses on microorganisms of interest to reflect human fecal pollution (*Escherichia coli* and *Enterococcus* spp.). Pollution by antibiotic substances in freshwater has been described near urban areas [24] but poorly studied in seawater. Further bacteriological studies into the marine environment are needed to understand the involvement of fecal flora contaminating seawater in the pathophysiology of pneumonia associated with drowning.

To reduce the risk of emergence of AmpC-hyperproducing *Enterobacteriaceae*, European guidelines discourage the use of third-generation cephalosporins as single-drug therapy against *Enterobacter* spp. [25]. When antibiotic treatment cannot be delayed, fourth-generation cephalosporin such as cefepime could be suggested as a first-line treatment and later downgraded according to bacteriological results. When direct examination can be rapidly obtained, cefepime may be kept specifically for Gram-negative bacilli, whereas Gram-positive cocci could be treated with amino-penicillin combined with a beta-lactamase inhibitor. The authors highlight that antibiotic treatments should always be tailored to local epidemiological microbial data.

Patients presenting with an AMC-resistant bacteria respiratory infection seemed to have a more severe initial clinical presentation (more cardiac arrest, longer resuscitation duration and higher drowning grade) and worse outcome (duration of mechanical ventilation, mortality in ICU), which was not associated with specific demographic characteristics (age, sex or comorbidity). Indeed half of them were less than 65 years and had never been hospitalized.

This point should be specifically addressed in a larger prospective study and a larger antibiotic spectrum discussed for patient with an initial severe clinical presentation notably after cardiac arrest and a high drowning grade.

Our results show a clear difference between seawater and freshwater drowning-associated pneumonia, with recent data promoting the use of a broad-spectrum antibiotic and even suggesting empirical antifungal therapy [5, 6]. Should this be confirmed in a future prospective study combined with environmental analysis, the water composition should define the antimicrobial strategy for empirical treatment.

### Limitations

This study represents a local population admitted to the two intensive care units with the biggest local recruitment of severe drowning. However, patients were all found in a similar coastal environment and mostly during the summer and spring. The marine bacterial ecology may change according to the season, location and human pollution, but the impact of such modifications is still unknown and can be minimal if the main mechanism involved is aspiration of the endogen flora of the patients. Indeed, seawater samples have not been described in our study because they were not considered to represent the water composition during the ten years of the study. Such samples could be analyzed in a future prospective study and be compared with respiratory sputum collected from patients during the same period.

Only 24/36 patients presenting with early pneumonia had a respiratory sample. This may affect the prevalence of the susceptible microorganisms observed in this study.

The absence of anaerobic bacteria found in the respiratory samples does not formally exclude their potential impact in the pathogenic process of pneumonia associated with seawater drowning. Indeed, these bacteria are fragile and require specific conditions and media for culture [26, 27]. Even if we cannot formally exclude anaerobic germs in this inhalation context, according to our results treatment with specific empirical antibiotic against these bacteria seems unnecessary.

Finally, the diagnosis of pneumonia is difficult for this population. An X-ray and biomarkers cannot be considered as a relevant tool to confirm or rule out diagnosis. Bacteriological samples may be of clinical, economical and of ecological interest.

## Conclusions

This cohort is the largest providing early respiratory samples from seawater drowning patients. Unlike recent data from freshwater drowning series, microorganisms found are mostly bacteria with a low rate of antibiotic resistance suggesting that drowning environment influenced previous observations. When empirical antibiotic therapy is required after seawater drowning, systematic broad-spectrum antibiotics appear unnecessary. Early respiratory sample collection should nonetheless be mandatory to avoid inappropriate therapy. Empirical cephalosporin could be proposed for the most severe patients or, if a direct examination can be rapidly performed, for Gram-negative bacilli, whereas patients with Gram-positive cocci could be treated with amino-penicillin combined with a beta-lactamase inhibitor.

## Abbreviations

ARDS: acute respiratory distress syndrome
MDR: multidrug resistant
ICD: International Classification of Diseases
ICU: intensive care unit
SAPS2: simplified acute physiology score 2
COPD: chronic obstructive pulmonary disease
AMC: amoxicillin–clavulanate
